# Prenatal, perinatal and parental risk factors for autism spectrum disorder in China: a case- control study

**DOI:** 10.1186/s12888-024-05643-0

**Published:** 2024-03-20

**Authors:** Jia-jia Yuan, Ya-nan Zhao, Xing-yu Lan, Yong Zhang, Rong Zhang

**Affiliations:** 1 Department of Neurobiology, School of Basic Medical Sciences, Peking University, Beijing, China; 2 Neuroscience Research Institute, Peking University, Beijing, China; 3 Key Laboratory for Neuroscience, Ministry of Education/National Health Commission, Beijing, China; 4 Autism Research Center of Peking University Health Science Center, Beijing, China; 5 School of Public Health, Peking University, Beijing, China; 6 China Center for Health Development Studies, Peking University, Beijing, China; 7 APEC Health Science Academy Peking Universities, Beijing, China; 8 Department of Integration of Chinese and Western Medicine, School of Basic Medical Sciences, Peking University, Beijing, China

**Keywords:** Autism spectrum disorder, Case control study, Risk factors, Prenatal, Perinatal, Parenting

## Abstract

**Background:**

Autism spectrum disorder (ASD) is heritable neurodevelopmental disorders (NDDs), but environmental risk factors have also been suggested to a play a role in its development. Prenatal, perinatal and parental factors have been associated with an increased risk of ASD in children. The aim of the present study was to explore the prenatal, perinatal, and parenting risk factors in children with autism spectrum disorder (ASD) from Beijing, China by comparing them with typically developing (TD) children.

**Methods:**

A sample of 151 ASD children’s parents who from rehabilitation institutions in Beijing were enrolled in this study, and an additional 151 children from kindergartens in Beijing were recruited as a control group (child age: mean = 4.4 years). TD children were matched according to age, sex and maternal education. We explored the maternal AQ (Autism Spectrum Quotient) scores (mean:19.40-19.71, no significant difference between two groups) to referring the genetic baseline. This study evaluated 17 factors with unadjusted and adjusted analyses.

**Results:**

Birth asphyxia was associated with a more than a thirteen-fold higher risk of ASD (adjusted odds ratio (*AOR*) = 13.42). Breastfeeding difficulties were associated with a higher risk of ASD(*AOR* = 3.46). Parenting influenced the risk of ASD, with low responding (LR) and harsh or neglectful parenting associated with a higher risk of ASD in offspring (*AOR* = 2.37 for LR, *AOR* = 3.42 for harsh parenting and *AOR* = 3.01 for neglectful parenting). Maternal fever during pregnancy was associated with a higher risk of ASD in offspring (*AOR* = 3.81).

**Conclusions:**

Many factors were associated with ASD in offspring. Further assessment is needed to elucidate the role of modifiable environmental factors to inform prevention strategies.

## Introduction

Autism spectrum disorders (ASD) are neurodevelopmental disorders characterized by impairments in reciprocal social interactions, communication, restricted interests and display of stereotyped behaviors [1]. During the past decades, numerous investigations have attempted to the causes of ASD. However, the mechanisms leading to the development of ASD remain unknown. Studies have shown that both genetic and environmental factors could lead to the ASD pathogenesis [2–3]. While genetic factors highly influence the odds of being diagnosed with ASD, a proportion of neurodevelopmental disorder causality has been attributed to environmental factors, especially nonshared environmental effects [4, 5]. Animal, human cell, and epidemiological studies suggest a wide range of environmental risks impact on neurodevelopment, which include prenatal, perinatal, postnatal and environmental factors [6].

Regarding the prenatal period, maternal fever during pregnancy is a risk factor for ASD in offspring [7]. Maternal exposure to toxins such as smoking and pollutants during pregnancy is also a risk factor for ASD [8–9]. Additionally, maternal exposure to stressful life events during pregnancy is a potential risk factor for ASD according to both animal experiments and human studies [10–12]. Several studies have focused on the perinatal period. The factors associated with the development of ASD include gestational age (GA), caesarian delivery prematurity, congenital malformations, low birth weight (LBW) and birth asphyxia [13–15]. Feeding difficulties might be a risk factor [16]. Regarding parenting, researchers have utilized different definitions of parental styles. The two important aspects of parenting are responsiveness and control [17]. Researchers have found that increased parental responsiveness facilitates child development, such as increases in expressive and receptive vocabularies [18–20]. Negative parenting is related to an increase in behavioral problems in both children with high and low genetic risks of ASD [21, 22].

While the general importance of environmental factors for ASD is undisputed, it remains unclear which factors may pose a specific risk and whether exposure to several factors may further increase the likelihood of ASD. The existing studies on the environmental risk factors of ASD have limitations. First, it is difficult to control genes to analyze environmental factors alone because of familial confounders, which are shared factors within a family, including both unmeasured shared environmental and genetic factors [5]. Environmental factors cannot be ruled out that they are driven by genetic links between exposure and outcome, and not by the environment itself. This has often been neglected in previous research on environmental factors [12, 15, 16]. Second, some of the factors have not yet been sufficiently studied. Few studies have examined postnatal or parenting influences [23–25]. The family experience of children in their early years is an environmental factor that cannot be ignored. Third, there is a lack of research under different cultural scenarios, most studies were conducted in North America and Europe, pointing to a global research bias [5]. The previous studies in China did not consider genetic and parenting factors [16, 24].

Therefore, the aim of the present study was to identify the prenatal, perinatal, parenting factors associated with ASD by comparing ASD children with typically developing (TD) children in consideration of genetic baseline. We hypothesize that 13 previously reported prenatal, perinatal risk factors and 4 parenting factors for ASD, in group of children with ASD and compared the empirical findings with those of a control group of TD children matched for age, sex and maternal education. Evidence for a range of environmental factors of potential casual significance in ASD is important, however, more genetically informed studies of good quality in the quest of the environmental causes of ASD is critical needed. We explore the maternal Autism Spectrum Quotient (AQ) scores to refer the genetic baseline, referring to previous studies [26, 27]. We conducted this study in the background of China, as different countries may vary in risk factors.

## Methods

### Participants

In order to increase the sample size as much as possible to improve explanatory power, 151 pairs of samples were included in the analysis. We recruited 151 parents of ASD children, who were followed up at four rehabilitation institutions in Beijing, China. Additionally, as a control group, 151 TD children’s parents were recruited and followed up at a kindergarten in Beijing, China. We believe the sample size is sufficient to complete the study, referring to previous relevant case-control studies [28, 29]. We conducted the survey in the form of face-to-face interviews with parents and electronic family questionnaires. All basic information and prenatal, perinatal and parenting information came from parental reports. To increase the accuracy of the information collected, we encouraged parents to consult the relevant documents and asked parents to add relevant details in the face-to-face interviews.

### Case ascertainment

This study included the parents of 3 to 6 years old children with ASD. We confirmed the diagnostic process of children from their parents. The children were diagnosed with ASD at one or more hospital and the hospital had diagnostic qualifications and followed a DSM-5 standard, not only through scale measurement but also via medical professional diagnosis. ASD children with comorbidities such as attention deficit hyperactivity disorder (ADHD), generalized growth retardation, and other diseases that affected behavior, such as malignant tumors and brain trauma, were excluded from the sample. As somatic and psychiatric comorbidities are frequent in neurodevelopmental disorders (NDDs) and significantly impact developmental mechanisms, it was necessary to control comorbidities in this study. Children in the TD group had normal intelligence, normal development, and no physical and mental illnesses, as confirmed by the teachers or school doctors.

These children matched the ASD cases by age, sex and maternal education and were randomly selected from the same administrative district kindergarten in Beijing, China. Because the control group might have had higher maternal education due to sampling location, we also matched children on maternal educational in this study. The AQ scores were used to preliminarily reflect the genetic status of the two groups of families.

### Pilot test

We designed a questionnaire and initially pilot tested it with a convenience sample. The questionnaire was modified according to the results obtained and was subjected to further validation procedures such as construct and reliability testing. For construct validation, percentage agreement and the content validity index were calculated and for reliability testing, internal consistency of test–retest results were assessed.

### Measures

The risk factors of interest were selected on the basis of recent empirical research on ASD. Specifically, they were as follows:

- Prenatal

Research into prenatal factors has focused on broad areas of psychiatric and neurological conditions, overweight status and inflammation, autoimmune reaction, and pregnancy-specific conditions. The questionnaire was designed based on probable risk factors for ASD (according to the literature) and those likely to be specific to the Chinese environment. Prenatal characteristics included poor maternal health during pregnancy and maternal exposure to stressful events. Poor maternal health included incidence of fever, diabetes, hypertension, anemia, vitamin deficiency and exposure to toxic substances/pollution during pregnancy. Maternal exposure to stressful events included major life changes (unemployment, divorce, or death of relatives), family violence and natural disasters.

b) Perinatal

We evaluated four perinatal risk factors: gestational age (GA), delivery method, neonatal status and postpartum maternal mental illness. GA was classified as 37–42 weeks, < 37 weeks and > 42 weeks. The delivery methods included natural delivery and cesarean birth. Neonatal status included healthy, birth asphyxia. Postpartum maternal mental health was added to assess maternal mental health after birth including depressive condition, anxiety condition, compulsive state and other mental disorders. Although breastfeeding difficulties do not only occur in the perinatal period, in order to distinguish between parenting behaviors, they are also considered as the characteristics of the perinatal period. In fact, most of the difficulties in breastfeeding occur between 1 day to 2 years after delivery. Breastfeeding difficulties include insufficient milk, poor milk flow, and children’s difficulty in sucking milk in this study.

c) Parenting

Regarding parenting, we assessed the frequency of touching or hugging, verbal or behavioral responsiveness and parenting style before the child was 2 years old. The frequency of touching or hugging their children was divided into “once a day or more” and “less than once a day”. Verbal or behavioral responsiveness was divided into “more” and “less” according to parental self-reports. The parenting style was divided into three groups: gentle, harsh and neglectful.

d) Others

Maternal age at the time of the child’s birth was classified as ≤ 34 years or > 34 years. The chosen age cutoff (34 years) for both parents was based on related studies [30, 31]. We also added the parental history of mental illness and chronic diseases (metabolic, immune or fertility problems) as control variables.

#### AQ scores

The AQ is a self-administered questionnaire that is used to identify autism spectrum–related traits in young people and adults [32]. It consists of 50 items organized into five subscales: social skill, attention switching, attention to detail, communication and imagination. A traditional case-control approach ignores the view that ASD is not just a spectrum within the clinical population, but that autistic traits are continuously distributed right through the general population [26]. The AQ is widely used to quantify autistic traits, which have been evaluated in the parents of individuals with ASD and in the general population. Some prior studies from Western cultures have shown that the parents of ASD children score higher than do the parents of TD children on the AQ score [26, 33]. Age, gender and SES might not associate with the total score of the AQ [34]. As the same genetic variants that put the child at risk for ASD may also lead to a broad phenotype that can be seen in close relatives of people with ASD, we control the AQ scores of their parents in this study. The AQ can be used to make stratification in genetics in previous studies [26, 27]. For example, the information from autistic traits by AQ scores were used to identify potential genetic variants in the pedigree and found that a greater number of Tier 1 genes are identified with increasing stringency of the AQ cut-offs [27]. The AQ score of parents in ASD group is not significantly higher than that in TD group in this study and it can be considered that there is not much significant difference in maternal genes between the two groups of parents.

### Statistical analysis

Statistical analysis of the data was performed using the SPSS version 22. All sociodemographic and perinatal characteristics were categorical and were expressed as frequencies (and percentages). The chi-square test was used to evaluate potential associations between the incidence of ASD and potential risk factors, while odds ratios (ORs) and 95% confidence intervals (CIs) were estimated as the measure of these associations. Maternal and child’s age was also expressed as the mean (standard deviation) and was compared between groups using a t test. A multivariate stepwise logistic regression model was constructed to explore the independent effects of child’s characteristics on the incidence of ASD. The multivariate regression was bidirectional. All tests were two-tailed and statistical significance was considered for *P* values < 0.05.

### Community involvement

In the research topic determination and questionnaire design, we consulted many clinical psychologists, neuropsychologists and developmental psychologists. At the same time, a large number of parents of autistic children participated in our interview when we designed the survey. But parents will not be involved in the recruitment and conduct of the study. After signing consents by the participants, they will be assessed for eligibility and data collection will begin. Dissemination of the general results (no personal data) will be made on demand.

## Results

### Socioeconomic and demographic factors

A total of 302 participants (*n* = 151 cases and *n* = 151 controls) were enrolled in this study. The average age of the children was 4.4 years, and boys accounted for 77% of participants. The mean ages of the mothers in the ASD and TD groups were 34.93 (SD: 3.92) and 34.88 (SD: 4.00) years, respectively. Approximately 90% of the mothers had a bachelor’s degree or above. There was no significant difference in child age or sex or maternal education, age, or birth age and AQ scores between the two groups (Table 1).

**Table 1:** Sociodemographic characteristics of ASD and TD children

	ASD (*N* = 151)	TD (*N* = 151)	*P* value
	M/N(SD/%)	M/N(SD/%)	
Child age	4.43(1.15)	4.42(1.12)	0.960
Child sex			
Male	116(76.82)	118(77.14)	1.000
Female	35(23.18)	33(22.86)	
Maternal age	34.93(3.92)	34.88(4.00)	0.783
Age of childbearing(mother)	30.40(3.96)	30.28(4.03)	0.923
Maternal AQ scores	19.40(5.66)	19.71(3.69)	0.569
Maternal education			
High school or below	14(9.27)	17(11.26)	0.979
Bachelor’s degree or above	137(90.73)	134(88.74)	

Abbreviations: ASD, autism spectrum disorder, TD, typically developing, AQ, Autism Spectrum Quotient

### Prenatal, perinatal and parenting characteristics

In the prenatal period, significantly higher frequencies of maternal fever (31.13% vs. 12.58%, *p* < 0.05) was observed in the ASD group than in the TD group. In the perinatal period, a higher incidence of birth asphyxia (9.27% vs. 0.66%, *P* < 0.05) and postpartum maternal mental illness (44.37% vs. 27.15%, *p* < 0.05) and feeding difficulties (12.58% vs. 3.97%) was observed in the ASD group than in the TD group. In the postnatal period, a higher incidence of LR (5.95% vs. 0.60%, *p* < 0.05), harsh parenting (18.54% vs. 5.96%, *p* < 0.05), and neglectful parenting (32.45% vs. 15.23%, *p* < 0.05) was observed in the ASD group than those in the TD group (Table 2).

**Table 2:** Prenatal, perinatal and parental factors of ASD and TD children (*n* = 302)

Variable	ASD (*N* = 151)	TD (*N* = 151)	*P* value
	N (%)	N (%)	
Prenatal			
Poor maternal health during pregnancy			
Fever	47(31.13)	19(12.58)	< 0.000
Diabetes	21(13.91)	15(9.93)	0.375
Hypertension	1(0.66)	4(2.65)	0.371
Anemia	11(7.28)	21(13.91)	0.091
Vitamin deficiency	4(2.65)	3(1.99)	1.000
Exposure to toxic substances/pollution	8(5.30)	9(5.96)	1.000
Maternal exposure to stressful events during pregnancy			
No	137(90.73)	146(96.69)	0.055
Yes	14(9.27)	5(3.31)	
Perinatal			
Gestational age			
37–42 weeks	143(94.70)	143(94.70)	1.000
<37 weeks	6(3.97)	6(3.97)	
>42 weeks	2(1.32)	2(1.32)	
Delivery			
Natural birth	109(72.19)	111(73.51)	0.897
Cesarean birth	42(27.81)	40(26.49)	
Neonatal status			
Healthy	135(89.41)	148(98.02)	< 0.000
Birth asphyxia	14(9.27)	1(0.66)	
Postpartum maternal mental illness			
No	84(55.63)	110(72.85)	< 0.000
Yes	67(44.37)	41(27.15)	
Feeding difficulties			
No	132(87.44)	145(96.03)	< 0.000
Yes	19(12.58)	6(3.97)	
Postnatal/Parenting			
Frequency of touching or hugging			
Once a day or more	143(94.70)	144(95.36)	1.000
Less than once a day	8(5.30)	7(4.64)	
Verbal or behavioral responsiveness			
High	158(94.05)	167(99.40)	< 0.000
Low	10(5.95)	1(0.60)	
Parenting behavior			
Gentle	74(49.01)	119(78.81)	0.001
Harsh	28(18.54)	9(5.96)	
Neglectful	49(32.45)	23(15.23)	

Abbreviations: ASD, autism spectrum disorder, TD, typically developing

### Risk factors for ASD (bivariate and multivariate analysis)

The bivariate analysis showed that 9 variables were significant in the first step. These variables were included in the multivariate logistic regression in the second step. In the second step, 8 variables showed a significant association with ASD (Table 3).

**Table 3:** Bivariate and multivariate logistic regression analysis result for variables

Variable	COR (95% CI)	AOR (95% CI)
Prenatal		
Poor maternal health		
Healthy	1.00	1.00
Fever	3.14(1.74–5.67) **	3.81(1.87–7.79) **
Diabetes	1.47(0.72–2.96)	1.87(0.79–4.44)
Hypertension	0.25(0.03–2.22)	0.28(0.02–3.31)
Anemia	0.49(0.23–1.05)	0.38(0.15–0.94) *
Vitamin deficiency	1.34(0.30–6.10)	0.88(0.15–5.21)
Exposure to toxic substances/pollution	0.88(0.33–2.35)	0.67(0.21–2.17)
Maternal exposure to stressful life events during pregnancy		
No	1.00	1.00
Yes	2.98(1.05–8.51) *	2.41(0.63–9.18)
**Perinatal**		
Gestational age		
37–42 weeks	1.00	1.00
<37 weeks	1.00(0.31–1.04)	1.01(0.24–4.37)
>42 weeks	1.00(0.14–7.20)	0.47(0.04–5.60)
Delivery		
Natural birth	1.00	1.00
Cesarean birth	1.07(0.64–1.78)	0.93(0.51–1.64)
Neonatal status		
Healthy	1.00	1.00
Birth asphyxia	15.33(1.99-118.11) *	13.42(1.26-143.29) *
Postpartum maternal mental illness		
No	1.00	1.00
Yes	2.14(1.32–3.46) *	1.21(0.67–2.20)
Feeding difficulties		
No	1.00	1.00
Yes	3.48(1.35–8.97)**	3.46(1.18–10.12)*
**Postnatal/Parenting**		
Frequency of touching or hugging		
Once a day or more	1.00	1.00
Less than once a day	1.15(0.41–3.26)	0.80(0.23–2.76)
Verbal or behavioral responsiveness		
High	1.00	1.00
Low	2.18(1.48–2.70)**	2.37(1.24–4.52)**
Parenting behavior		
Gentle	1.00	1.00
Harsh	5.00(2.24–11.19)**	3.42(1.40–8.36) **
Neglectful	3.43(1.93–6.08) **	3.01(1.59–5.70) **

Abbreviations: ASD, autism spectrum disorder, TD, typically developing; COR = crude odds ratio, AOR = adjusted odds ratio. Results were from multiple logistic regression adjusted for child age/sex, maternal age and AQ scores

*:*p* < 0.05; **:*p* < 0.01

Specifically, maternal fever during pregnancy was significantly associated with offspring ASD, with an *AOR* of 3.81 *(95% CI*: 1.87–7.79). Birth asphyxia was a strong risk factor for ASD in this study. The risk of ASD among children who experienced birth asphyxia was 13.42 times higher than those who did not experience birth asphyxia, with an *AOR* of 13.42 *(95% CI*: 1.26-143.29). The risk of ASD among mothers who experienced feeding difficulties was 3.46 times higher than those who no such experience with an *AOR* of 3.46 *(95% CI*: 1.18–10.12). In regard to parenting behavior, LR was significantly associated with a greater likelihood of ASD, with an *AOR* of 2.37 (95% CI: 1.24–4.52). Parents had different ways of comforting a crying child. Compared with gentle parenting, harsh or neglectful parenting were more likely to result in children with ASD (*AOR* = 3.42; *95% CI*: 1.40–8.36 for harsh parenting; *AOR*: 3.01; 95% *CI*: 1.59–5.70 for neglectful parenting) (Table 3).

In this study, we did not find any significant association of diabetes, hypertension, vitamin deficiency, exposure to toxic substances/pollution, gestational age, delivery method or frequency of touching or hugging with ASD.

## Discussion

This is one of the pioneering studies in China that focus on the risk factors of ASD in offspring. Previous studies have reported significant associations of prenatal, perinatal and neonatal risk factors with ASD, but few study has been conducted in China and make parenting as a risk factors. More importantly, the lack of attention to genes makes it difficult to explain environmental factors. In this study, we have conducted a case–control study, matched on age, sex and maternal education to investigate prenatal and perinatal and parenting risk factors for ASD in China. The main strengths of this investigation include: (a)a comprehensive list of risk factors is examined, (b) parenting factors are included and (c) The AQ score are used to compare the autistic traits in parents, partially controlling the influence of genes. Specifically, there were several main findings from this study.

First, birth asphyxia increased the risk of ASD. According to our findings, newborns who experienced birth asphyxia were 13.42 times more likely to be subsequently diagnosed with ASD than children who did not experience birth asphyxia. A strong relationship between birth asphyxia and ASD has also been confirmed by other studies. For example, an Indian study found that children who experienced birth asphyxia were 10.63 times more likely to develop ASD than other children [15], which was highly consistent with our results. Previous research found that fetal hypoxia is one of the manifestations of fetal distress [10] and that fetal distress increases the risk of late brain dysplasia. In fact, oxidative stress is one of the strongest environmental and perinatal factors believed to contribute to ASD [8]. The findings of this paper once again emphasize the negative impact of birth asphyxia.

Second, maternal fever during pregnancy was significantly associated with offspring ASD. Previous studies have reported a positive association between maternal fever and offspring ASD [35, 36]. A mechanism proposed to underlie this relationship is the development of maternal antibodies against fetal brain tissue; this maternal immune activation could lead to neurodevelopment abnormalities in children [37]. Some studies have investigated whether the use of medication to control fever symptoms affects the relationship between fever and offsprings’ ASD [37, 38]. In this study, most of the mothers who experienced fever used medication, but we lacked the necessary information to disentangle these effects. Further research is necessary to elucidate the relationship between fever and ASD.

Third, breastfeeding difficulties were associated with a higher risk of ASD. Previous studies have also found a positive correlation between less breastfeeding and the risk of ASD in offspring [16, 39]. Breastfeeding during the critical phase of the child’s development plays a pivotal role in immune and neural development [40, 41]. The components of breast milk such as IgA, transforming growth factor-β,and lactoferrin stimulates intestinal host defenses as well as prevents inflammation [42]. What’ more, breastfeeding is critical for forming an emotional connection with their baby [43]. Breastfeeding difficulties may not only cause nutritional deficiencies in children, but also lead to poor bonding between the mother and child, resulting in poor sensory stimulation in the child [44]. In addition, breastfeeding issues are linked to genetic factors [45]. For example, infants diagnosed with ASD in their latter phases may face challenges throughout infancy, making it more difficult for the mothers to breastfeed them. Mothers of an autistic children may have autistic traits, such as sensory sensitivities or symptoms of other neurodevelopmental disorders such as ADHD, which would make it more difficult for them to breastfeed their infant. All of these factors might contribute to the pathogenesis of ASD.

Fourth, parental behavior greatly impacts on child development. Some parental behaviors significantly increase the risk of ASD in children. In this study, we found that LR to child needs was significantly positively associated with the risk of ASD. Previous research found that maternal sensitivity and attentiveness to child signals enhanced the child’s intrinsic motivation to learn [46]. Caregivers’ verbal responsiveness significantly influences child language development [46, 47] and the development of skills, such as joint attention [47]. We did not disentangle verbal and nonverbal responsiveness in this study. Caregiver responsiveness measures themselves typically combine these dimensions; moreover, we did not examine whether these dimensions had separate or additive effects [18]. Similarly, neglectful parenting, such as LR, is a parental style with low warmth and lack of attention. We found that neglectful parenting resulted in a higher risk of ASD than gentle parenting. Previous studies have found that neglectful parenting predicts externalizing behavior problems in offspring [48, 49], consistent with the conclusion of this study. If we do not control for AQ scores, the relationship between parental behavior and ASD outcomes remains inconclusive. Because parents with higher levels of autistic traits had more parenting difficulties [50, 51], the genes are still at play in the end. Due to the control of AQ scores, behavioral factors can be independently assessed in this study.

Fifth, harsh parenting increased the risk of ASD in children. Harsh parenting usually means a lack of warmth. Parental warmth, defined by displays of physical and verbal affection, facilitate child development [52]. Harsh parenting is worse than neglectful parenting in terms of the risk of ASD. Compared with gentle parenting, harsh parenting was associated with a more than fourfold higher risk of ASD. Similar studies have found that harsh discipline, assertion of power and withdrawal of love generally have negative consequences for children, leading to aggressive behavior and emotional problems [22, 53].According to emotional security theory, children desire to feel secure in their environment [54]. Negative parenting behaviors, such as low warmth, rejection and harsh discipline, may threaten feelings of security and lead to dysregulation of the stress response system. Moreover, negative parenting impacts children’s biological stress response systems, which are often measured in terms of cortisol [55]. Low parental warmth could lead to dysregulation of cortisol levels [56], which has been linked to adverse psychological consequences [57]. Recently, factors such as parental warmth, sensitivity, and communication have been examined as influential factors that shape the cognitive and social skills of individuals with ASD. Our finding strongly suggests a link between these nonresponsive or harsh parenting behaviors and adverse child outcomes. Caregiver behavior is a key part of the child’s environment and might be a critical mechanism underlying changes in child development, although additional research is needed to clarify the specific mechanisms. In our study, more than 90% of parents provided more touching and hugging of children and high verbal and behavioral responsiveness. More harsh and neglectful parenting was more likely to lead to ASD in children, although the heightened risk may not only relate to parenting behavior but also genetics. Further research is needed for more in-depth analysis. Our research group is conducting animal experiments that have provided preliminarily confirmation that the negative effects of parenting behavior on offspring are independent of genetics.

Sixth, maternal exposure to stressful life events during pregnancy was a risk factor for ASD. Many studies have suggested that negative or stressful life events have a major psychological impact on pregnant mothers, which increases the risk of ASD in their offspring [10, 11, 58]. The assessed stressful events usually include natural disasters [10], family discord [58] and bereavement [59]. Our study expanded this category to assess natural disasters, major life changes (unemployment, divorce, death of relatives), and family violence. We found that the risk of ASD in offspring among mothers who experienced stressful life events during pregnancy was more than two times higher than that among mothers who did not experience stressful life events during pregnancy, supporting previous findings. One explanation is that prenatal stress decreases exposure to prenatal testosterone [60], a hormone that exerts a protective effect on fetal development.

Above all, the ASD risk factors have been overidentified as many studies may be detecting associations with hypothesized ASD without adequately addressing confounding of other known or suspected risk factors. In this study, we controlled the AQ scores. This makes our discovery more persuasive. Furthermore, due to factors such as easier access to medical and health services, parents with higher socioeconomic classes were more likely to have their children diagnosed, resulting in disparities in prevalence based on socioeconomic status [61, 62], the overall proportion of mothers with higher education in this study sample was higher than the national average. Similarly, mothers of TD children were matched, decreasing the impact of education on the results.

### Limitations

We conducted a case‒control study to identify risk factors for ASD. Our study provides important country-specific evidence for this field of study. However, this article has some limitations. First, parenting behavior is not an exogenous variable and can be affected by many factors, such as heredity, race, culture, family income, and education level. Parental psychopathology is associated with negative parenting behavior such as neglect, aggression, and poor emotion socialization. The effects of genetic and environmental factors are complex. To better determine the impact of parenting style on child outcomes, we need to further control parenting behavior. Second, despite reported associations between environmental factors and ASD, the evidence to support a causal relationship remains insufficient. Although we used maternal AQ to control the genetic background, it is unclear how generalizable our findings are. Paternal AQ should have been studied, although the existing studies are mainly focused on mothers and maternal AQ scores are more sensitive [63, 64]. ASD symptoms are multifaceted, and parents’ AQ may not be highly sensitive to predicting parental ASD symptoms. More comprehensive genetic identification is required in future studies. Third, the collection of information regarding risk factors by parental self-report is a technical limitation. Mothers were asked to recall the experiences such as postpartum mental health or breastfeeding problems, which might result in recall bias or error. Obtaining more objective indicators such as APGAR score in perinatal [65] is critical for future improvement. Prospective cohort studies may also be an alternative solution for future studies to solve this issue. Fourth, the maternal education level in both groups is high, which limits the generalizability of the findings. Cautions should be taken when extrapolating it. Fifth, although we have provided several classifications of breastfeeding difficulties, they may not be comprehensive and there may be overlap between options. We have not conducted in-depth discussions. In the future, a more detailed analysis of breastfeeding is needed. Sixth, postpartum mental diseases such as depression, anxiety, and obsessive behavior are all psychiatric terminology. Using descriptions like “low mood” and “excessive worry” may be more appropriate because the respondents will understand them better. Seventh, the survey method yields insufficient objective basis for diagnosis, which is an issue in most questionnaire surveys. In the future, we will try to sample patients with objective diagnoses, such as hospital medical records, to improve the accuracy of ASD samples.

## Conclusion

Our study categorically implicates many prenatal, perinatal and parental characteristics as risk factors for ASD, adding important country-specific information to the literature. Out of all the factors analyzed, birth asphyxia, breastfeeding difficulties, lower parental responsiveness, harsh or neglectful parenting and maternal fever during pregnancy were associated with a greater than 2-fold increase in the risk of ASD. These findings highlight the necessity of additional focused investigations to disentangle the effects of genetic and environmental factors. Even if contributing developmental liabilities prove to be secondary, new understanding of the role of environmental influences especially the parenting, offers additional hope for novel strategies to ameliorate the severity of the condition in affected children. Our findings suggest that perinatal care may be critical for reducing the incidence of ASD. Identification of risk factors can help develop plans to detect and prevent early feeding difficulties and inappropriate parenting behaviors.

## Data Availability

The data that support the findings of this study are available on request from the corresponding author, RZ, upon reasonable request.

## Abbreviations

ADHD: Attention deficit hyperactivity disorder
AOR: Adjusted odds ratio
ASD: Autism spectrum disorders
AQ: Autism spectrum Quotient
CIs: Confidence Intervals
DSM-5: Diagnostic and Statistical Manual of Mental Disorders, 5th edition
GA: Gestational age
LBW: Low birth weight
LR: Low responding
NDDs: Neurodevelopmental disorders
ORs: Odds ratios
TD: Typically developing
